# An Appraisal on the Value of Using Nutraceutical Based Senolytics and Senostatics in Aging

**DOI:** 10.3389/fcell.2020.00218

**Published:** 2020-04-03

**Authors:** Amanpreet Kaur, Salvador Macip, Cordula M. Stover

**Affiliations:** ^1^Department of Respiratory Sciences, University of Leicester, Leicester, United Kingdom; ^2^Mechanisms of Cancer and Ageing Laboratory, Department of Molecular and Cell Biology, University of Leicester, Leicester, United Kingdom; ^3^Faculty of Health Sciences, Universitat Oberta de Catalunya, Barcelona, Spain

**Keywords:** senescence, senolytics, senostatics, nutraceuticals, aging

## Abstract

The average human life expectancy has increased globally, and continues to rise, owing to the substantive progress made in healthcare, medicine, sanitation, housing and education. This ultimately enriches society with a greater proportion of elderly people. Sustaining a healthy aged population is key to diminish the societal and economic impact of age-related infirmities. This is especially challenging because tissue function, and thus wellbeing, naturally progressively decline as humans age. With age increasing the risk of developing diseases, one of the therapeutic options is to interfere with the molecular and cellular pathways involved in age-related tissue dysfunction, which is in part caused by the accumulation of senescent cells. One strategy to prevent this could be using drugs that selectively kill these cells (senolytics). In parallel, some compounds have been identified that prevent or slow down the progression of senescence or some of its features (senostatics). Senolytic and senostatic therapies have been shown to be efficient *in vivo*, but they also have unwanted dose-dependent side effects, including toxicity. Important advances might be made using bioactive compounds from plants and foods (nutraceuticals) if, as is proposed, they offer similar effectiveness with fewer side effects. The focus of this review is on the use of nutraceuticals in interfering with cellular senescence.

## Introduction

Aging is the time-related deterioration of physiological functions necessary for survival and fertility (Gilbert, 2000). On a cellular level, aging is accompanied by the accumulation of cells that adopt a specific phenotype, known as senescence, a process which is conserved across species. Senescence is mainly characterized by an irreversible cell cycle arrest and the secretion of a group of factors, known as the senescence-associated secretory phenotype (SASP), whilst maintaining metabolic activity (Wiley and Campisi, 2016). Over time, senescent cells accumulate in all tissues and organs, contributing to their functional deterioration (van Deursen, 2014). The permanent cessation of cell proliferation is seen as a way to avoid neoplastic outgrowth, which means that in this context, senescence can be considered a tumor supressor mechanism (Dimri, 2005). For growth arrest to take place, senescent cells increase the expression of cyclin dependent kinase inhibitors (CDKIs) expression such as p21, p16 or p27 (Alcorta et al., 1996; Hernández-Segura et al., 2018).

The SASP, which is capable of promoting tissue repair and regeneration, creates a microenvironment that can drive neighboring cells into proliferation or even senescence, thus contributing to the disruption of tissue homeostasis (Rodier and Campisi, 2011). Therefore, with beneficial attributes early in life and detrimental consequences late in life, the senescence phenomenon is as an example of so-called antagonistic pleiotropy (Rodier and Campisi, 2011; Giaimo and d’Adda di Fagagna, 2012).

Due to general lack of specificity, multiple markers have to be considered and validated in conjunction for the identification of senescent cells. These markers include, but are not limited to, senescence associated beta-galactosidase staining (SA-β-Gal), cell cycle arrest, morphological and nuclear changes, and expression of certain proteins (Hooten and Evans, 2017). It is important to differentiate senescent cells from quiescent and terminally differentiated cells such as neurons, macrophages and muscle cells (Rodier and Campisi, 2011). Quiescent cells retain the ability to re-enter the growth cycle when stimulated with growth signals and are key to tissue regeneration by maintaining a constant state of balanced proliferation (Yao, 2014).

## Causes of Cellular Senescence

### Replicative Senescence

Hayflick and Moorhead first observed senescence in somatic cells serially passaged in culture (Hayflick and Moorhead, 1961). Normal cells undergo multiple mitotic cell divisions, eventually reaching a limit at which their proliferative capabilities are lost. This is now referred to as replicative senescence. Human fibroblasts, the cell type originally described, had a limit of around 50 passages. Since then, other forms of senescence have been discovered and applied in models of *in vitro* and *in vivo* research.

Most types of senescence are largely triggered through the DNA damage response pathways (DDR) (Fumagalli et al., 2014). The DDR consists of upstream components such as ATM (Ataxia telangiectasia mutated) and ATR (Ataxia telangiectasia and Rad3 related) kinases, which activate cell cycle checkpoint proteins CHK1 and CHK2. Eventually, cyclin dependent kinases (CDKs) are inhibited to arrest the cell cycle (Jackson and Bartek, 2009). In senescent cells, the main proteins involved in proliferative arrest are CDKIs p21 and p16. These are found to be overexpressed and are often used as markers for cell cycle arrest and senescence.

In replicative senescence, activation of the DDR is triggered through the shortening of telomeres. The change in DNA content following loss of telomeric length is recognized as damage, resulting in the initiation of the DDR response (Serrano and Muñoz-Espín, 2014). Telomeres are complexes composed of proteins and nucleotides of TTAGGG repeats at the ends of eukaryotic chromosomes that are considered protective structures (Bernadotte et al., 2016). When a cell divides, chromosomes are replicated and telomeres shorten in length with each cell division. An increase in the presence of telomere associated foci is seen in connection to aging (Hewitt et al., 2012). Loss of telomeric length has been suggested to be the biomarker of choice in replicative senescence (Bernadotte et al., 2016). Of note, lifestyle choices such as smoking, diet, stress and exercise can have an effect on telomere attrition (Shammas, 2011). Telomere attrition occurs in cells in which the expression of telomerase is repressed, as is the case in adult human somatic cells (Calado and Dumitriu, 2013). By contrast, mice regulate telomere length and telomerase activity differently from humans and are therefore inherently limited in studies of replicative aging (Zhang et al., 2016).

### Stress-Induced Premature Senescence (SIPS)

DNA single and double strand breaks (induced by exposure to radiation, overexpression of oncogenes, oxidative stress, etc.) result in the activation of the DDR and can eventually lead to a stress-induced premature senescence (SIPS), which is independent of telomere length (Boothman and Suzuki, 2008). *In vitro* SIPS models have been established using oxidative stress, ionizing radiation or DNA damage causing agents such as bleomycin (González-Hunt et al., 2018).

Overexpression of the *ras* oncogene in human and rodent cells was found to elicit a phenotype similar to cellular senescence, supporting the fact that senescence is a tumor-suppressor mechanism. Since then, other oncogenes have also been found to induce senescence including; Raf, c-Myc, Akt and E2F3 (Serrano et al., 1997; Astle et al., 2011; Qian and Chen, 2012; Ko et al., 2018).

*In vivo* models of SIPS include accelerated aging of mice as a result of gene defects. For example, mutations in the *LMNA* gene of mice can result in the onset of Hutchinson-Gilford progeria syndrome, deficiencies in *nfkb1* can lead to early aging phenotype in middle-aged mice and WRN protein deficient mice develop Werners syndrome, a disease of premature aging (Kõks et al., 2016). Recent models include the syngeneic transplantation of senescent cells in mice, leading to signs of physical dysfunction (Xu et al., 2018). These models have become the basis for senescence research, specifically for the testing of potential anti-aging drugs.

### CHARACTERISTICS OF CELLULAR SENESCENCE

**FIGURE 1:**
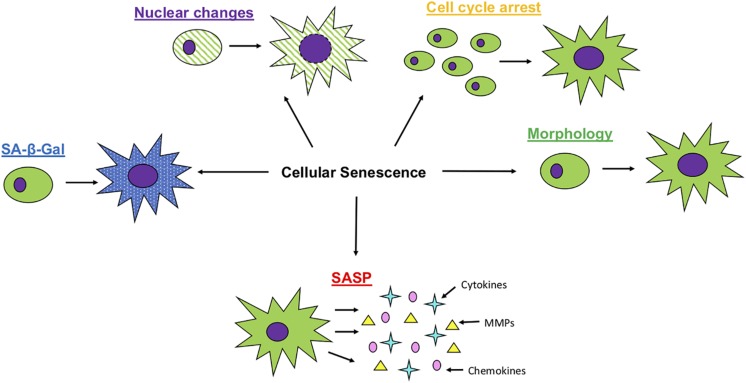
Characteristics of Cellular Senescence. Senescent cells will undergo multiple changes, with morphology becoming larger and flatter, an increased expression of SA- β -Gal activity, loss of nuclear membrane integrity, cease in cell proliferation and the production of SASP, including an increased expression of matrix metalloproteases (MMPs), cytokines and chemokines.

As outlined below and illustrated in Figure 1, senescent cells display changes in morphology, increases in certain cell cycle related proteins, nuclear changes and the presence of SASP.

### Morphology

Senescent cells in culture have a characteristic morphology that makes them distinguishable from proliferating cells (Dimri, 1995). Once in senescence, cells become flatter and the nucleus becomes enlarged. The cause for these morphological changes is not fully understood but the increase in cell size may be associated with the process of cell cycle arrest, where the imbalance of DNA: cytoplasm ratio in itself may contribute to aging (Neurohr et al., 2019).

### SA-β-Gal Activity

The cytoplasmic content of senescent cells also changes, with an increase in lysosomal SA- β-Gal activity. This enzyme is thought to exist in cell lysosomes, which are known to increase in size as cells age. Therefore, a high SA- β-Gal activity would be expected in senescent cells. However, this is not considered to be exclusive, as it can also be found in other cell types of non-senescent nature and in some cases may not be established in senescence (Dimri, 1995; Lee et al., 2006; Huang and Rivera-Pérez, 2014).

### Cell Cycle Arrest

The CDK/Cyclin complexes are necessary to progress through the cell cycle. In senescent cells, this interaction between cyclins and CDKs is blocked by CDKIs (Blagosklonny, 2011). The *INK4A* gene encodes the p16 protein, which inhibits the Cyclin D/CDK4/6 complex to stop the progression of G1 phase to S phase (Jingwen et al., 2017). It is often used as a marker for senescent cells due to its increased levels of expression. The *CDKN1A* gene encodes p21, which inhibits the Cyclin E/CDK2 and Cyclin B/CDK1 complexes (Jingwen et al., 2017). The inhibitor is commonly used a marker as well, but is less specific, since it can be upregulated in other situations (Hernández-Segura et al., 2018).

### Nuclear Changes

In addition to the increase in nuclear size, senescent cells have changes to the nuclear lamina, which impact on nuclear stability (Hernández-Segura et al., 2018). The nuclear lamina is made up of lamin proteins, which are type V intermediate filaments, and is disassembled and reassembled with every mitotic division (Freund et al., 2012). Lamin B1 is expressed differentially between pre-senescent and senescent cells: in various primary human fibroblast cell lines, a decrease of protein and mRNA expression of Lamin B1 can be observed, suggesting its use as a senescent cell biomarker (Freund et al., 2012).

Changes in chromatin structure are also exhibited in senescent cells. Heterochromatin becomes more prominent, with areas of nucleus having denser DAPI (diamidinophenylindole) stained foci that have been called senescence associated heterochromatin foci (SAHFs) and may be involved in gene silencing of proliferation genes (Aird and Zhang, 2012). These SAHFs can also be distinguished with specific epigenetic markers, such as H3K9me3, H3K27me3, and macroH2A, a facultative heterochromatin histone variant (Parry and Narita, 2016). DNA methylation patterns change during the process of aging as well, with hypomethylation in heterochromatic regions and focal hypermethylation in gene-associated cytosine-guanine islands (CGIs) (Ciccarone et al., 2018). Histone modifications can cause repression of certain genes, silencing those involved in cell cycle progression.

### SASP

The SASP consists of a collection of molecules secreted by senescent cells that shape the microenvironment. These molecules include proteases and growth factors, chemokines and cytokines such as IL-6, IL-8 and IL-1β (Borodkina et al., 2018). Interleukins take part in activating signaling pathways such as Jak/STAT, PI3K, and MAPK (Garbers et al., 2013). Proteases, such as matrix metalloproteases (MMPs) and serine proteases, are involved in the development and regeneration of the extracellular matrix. The inhibitors of these proteins and signaling molecules are also secreted by senescent cells to regulate their functions (Borodkina et al., 2018), so the net effect is dependent on this balance. Although the SASP works to promote tissue repair and regeneration and encourages immune surveillance, it can also be detrimental to health. For example, through paracrine induction of epithelial to mesenchymal transition and attraction of immunosuppressive immune cells, it can contribute to tumorigenesis and metastasis in cancer (González-Meljem et al., 2018).

## Chemical Approaches to Regulate Senescent Cell Accumulation and Activity

The characterization of compounds that could counteract the process of senescence has recently become a priority, given its immense therapeutic potential. These anti-senescence compounds are divided into two categories: senolytics and senostatics. Senolytic drugs are agents that selectively induce apoptosis of senescent cells. They were first discovered by Zhu et al. (2015), in whose work the combination of quercetin and dasatinib showed potential apoptotic activity in eliminating senescent cells (Zhu et al., 2015). On the other hand, senostatics are drugs capable of interfering with the progression of cells entering senescence or modulate their activity by reducing SASP generation (Short et al., 2019).

Senolytics can work by overriding anti-apoptotic pathways in senescent cells (Short et al., 2019). One of the most well studied senolytics is the combination of dasatinib and quercetin. Dasatinib is an established anti-cancer drug that, when administered in mice alongside quercetin, selectively eliminates senescent cells, leading to improved physical function and increased survival *in vivo* (Xu et al., 2018). This combination is now being tested as part of clinical trials to investigate its potential in patients with idiopathic pulmonary fibrosis, with preliminary results already showing improvements in cardiopulmonary function (Justice et al., 2019). Many compounds are under investigation for their potential senolytic properties. Other synthetic senolytic drugs include BCL inhibitor ABT-737, Panobinostat, HSP90 inhibitors or cardiac glycosides (Yosef et al., 2016; Fuhrmann-Stroissnigg et al., 2017; Samaraweera et al., 2017; Xu et al., 2018; Guerrero et al., 2019; Triana-Martínez et al., 2019).

Senostatics can work by inhibiting paracrine signaling or by counteracting the effects of the SASP in senescence (Short et al., 2019). They can also prevent the emergence of senescent cells by blocking fundamental steps of the effector mechanisms of the phenotype, such as activation of the p53 pathway (Althubiti et al., 2016). Examples of senostatic that have proven to be effective therapies in mouse models include rapamycin and metformin (Short et al., 2019). Rapamycin is a naturally derived antibiotic with additional anti-fungal and immunosuppressant properties. The macrolide compound is an mTOR inhibitor and it delays the progression of senescence and improves health in animal models (Blenis et al., 2014; Wang et al., 2017). Metformin, originally derived from *Galega officinalis* also known as Goat’s Rue (Bailey, 2017), is a well-established anti-diabetic drug that, has previously been shown to inhibit the SASP by interfering with the NFκB pathway (Moiseeva et al., 2013).

## Nutraceutical-Based Senolytics and Senostatics

**FIGURE 2:**
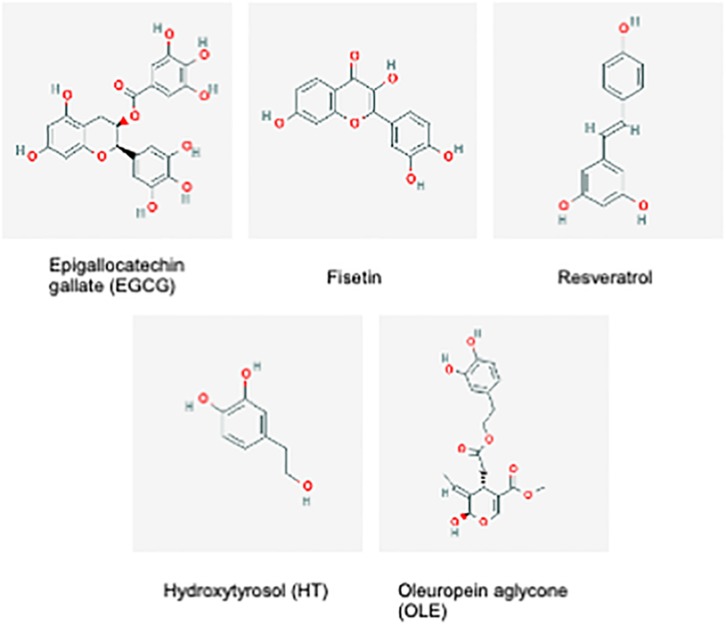
Chemical structures of Epigallocatchin gallate (EGCG), Fisetin, Resveratrol, Hydroxytyrosol (HT) and Oleuropein aglycone (OLE). These compounds consist of either phenolic of polyphenolic alcohol groups (National Center for Biotechnology Information, 2019a,b,c,d,e).

Synthetic compounds with senolytic or senostatic properties can be effective, however, they are not specific, and systemic side effects can be severe and deleterious to healthy cells (Malavolta et al., 2018). Hence, a movement toward the research of natural based compounds (nutraceuticals) with potential anti-senescence properties has begun. Nutraceuticals are bioactive compounds derived from food, including plant material, with physiological benefits in the prevention or treatment of disease (Rafieian-Kopaei et al., 2014). For instance, polyphenols, found in high abundance in plants, are bio-active compounds with anti-oxidant and anti-inflammatory properties making them potential senostatics by negating the pro-oxidant and pro-inflammatory signaling of senescent cells (Gurău et al., 2018). The aim remains to find potential anti-aging therapies that are effective but exhibit minimal side effects, and some natural plant-based compounds could fit this criterion. Below, we will discuss studied examples of nutraceuticals that could act as senolytics or senostatics as illustrated in Figure 2.

### Olive Phenols

The olive oil plant contains high amounts of phenolic compounds, which exhibit potential beneficial properties regarding cardiac health, cancer protection and antimicrobial effects (Tuck and Hayball, 2002; Marković et al., 2019). *In vitro*, two olive phenols called hydroxytyrosol (HT) and oleuropein aglycone (OLE) have shown to counteract senescence via significant reductions in SA-β-Gal staining, p16 levels and SASP levels in pre-senescent human lung fibroblasts (MRC5) and neonatal human dermal fibroblasts (Menicacci et al., 2017). HT has also been investigated in a UVA-induced senescence model in human dermal fibroblasts (HDFs) and significant reductions in SA-β-Gal positivity and mRNA expression levels for SASP related genes were observed after treatment (Jeon and Choi, 2018).

OLE reduces oxidative stress and inhibits mTOR, which is a key modulator in aging (Johnson et al., 2013; Sun et al., 2017). However, there are no data yet on the potential effects of OLE on organismal ageing. During the process of aging, the accumulation of misfolded and damaged proteins takes place, and these are degraded and removed through proteasome activity (Sáez and Vílchez, 2014). OLE has shown to improve proteasome activity, thereby delaying senescence in human fibroblasts. Furthermore, continuous OLE treatment of early passage human embryonic fibroblasts was shown to reduce ROS levels, curb the progression of the senescence phenotype by abating the changes in morphology seen in senescence, and lower cell mortality (Katsiki et al., 2007; Giovannelli, 2012; Menicacci et al., 2017).

### Green Tea Catechins

Catechin is a tannin found in green tea that has high antioxidant properties. As a polyphenol, it exists in multiple forms, including Epigallocatechin gallate (EGCG), which is found abundantly in tea leaves (Unno, 2016). Research investigating the effects of EGCG against replicative senescence in primary cells (rat vascular smooth muscle cells, HDF (human dermal fibroblasts) and human articular chondrocytes), has shown the potential senostatics effects of the nutraceutical (Han et al., 2012). SA-β-Gal staining of HDF cells treated with EGCG at early and late passages showed fewer positive cells in treated than controls (Han et al., 2012). Moreover, p53 was shown to be significant reduced in EGCG treated cells (Han et al., 2012). However, cell cycle analysis of HDFs with and without EGCG treatment showed that treated cells had a similar percentage of cell in S phase as control cells.

EGCG was found to suppress premature senescence in preadipocytes, with treated cells showing significant downregulation of ROS, SASP and p53 mediated cell cycle arrest, in addition to the suppression of the anti-apoptotic protein BCL-2 and an increase in cell death (Kumar et al., 2018). This suggests that EGCG could have senolytic properties.

The effect of green tea catechins in aging was studied using a senescence accelerated mouse model (SAMP_10_), which exhibits a short life span, cerebral atrophy and cognitive dysfunction (Unno et al., 2006). Between the ages of 1 and 15 months, mice were fed 35 mg/kg/day of green tea catechins. Analysis of blood and brain tissue found a decrease in oxidative DNA damage in the catechin-fed mice along with higher memory retention (Unno et al., 2006). A study investigating the effects of EGCG in healthy rats showed a median increase of lifespan by 8–12 weeks compared to controls. There was a significant decrease in liver and kidney IL-6 and ROS levels of the EGCG treated group, both of which are known to be upregulated with increasing age (Niu et al., 2013). This shows the potential beneficial systemic effects of EGCG in various organs.

However, an Intervention Testing Program was carried out, in which green tea extract was administered to a genetically heterogenous mouse model from a young age. Results showed no significant changes to lifespan (Strong et al., 2012). Although, the limited research of EGCG in connection to aging does not allow for definite conclusions, it has been widely used as a cosmetic for its potential skin protective effects its widely researched as a cosmetic additive having shown skin protective effects (Kim et al., 2018) and is available commercially in the form of green tea extract tablets.

### Fisetin

Fisetin is bioactive flavonol molecule found in fruits and vegetables such as cucumber, apple, grape and onions, with the highest concentration being found in strawberries (Khan et al., 2013). It has established antioxidant, apoptotic and anti-proliferative qualities (Khan et al., 2013). Application of 5 μM Fisetin for 48 h led to a significant reduction in SA-β-Gal positivity in oxidative-stress induced senescent murine embryonic fibroblasts (Yousefzadeh et al., 2018b). Progeroid Ercc1^–/Δ^ and aged wild type mice receiving a diet supplemented with fisetin for two 14-day periods over 14 weeks at a concentration of 60 mg/kg/day had significantly lowered p16 expression in adipose tissue, in addition to decreased SASP. Naturally aged C57BL/6 mice treated orally at 22–24 months with 100 mg/kg fisetin for 5 days showed a reduction in senescent cells in white adipose tissue (Yousefzadeh et al., 2018b). This result was mimicked in human adipose tissue explants that had been treated with fisetin and cultured *ex vivo*, showing reduction in SASP markers, IL-6, IL-8, MCP-1, and SA-β-Gal activity (Yousefzadeh et al., 2018b). Additionally, fisetin treatment at 85 weeks of age significantly prolonged life-span of these mice by an additional 3 months.

This research has given rise to the Alleviation by Fisetin of frailty, Inflammation and related measures in Older Adults (AFFIRM-LITE) clinical trial. Currently in the recruiting phase, the trial hopes to recruit 40 participants between the ages of 70–90 to take an oral 2-day dose of a placebo or fisetin at 20 mg/kg/day with analysis focusing on markers of frailty, inflammation, insulin resistance and bone metabolism (Clinicaltrials.gov, 2019a).

### Resveratrol

Resveratrol is a stilbene polyphenol commonly found in pigmented fruits and vegetables, such as grapes and berries (Risuleo, 2016; Salehi et al., 2018). Biological activities of resveratrol include being antitumor, phytoestrogenic, antioxidant and antiviral (Risuleo, 2016). In senescence, resveratrol has been investigated for its effects on WI-38 human fibroblasts and HT-1080 cells with inducible ectopic p21. With the application of resveratrol at 50 μM, the senescent morphology of both SIPS model-based cell types was prevented, suggesting a senostatic effect. Moreover, at these concentrations, there was a 2-fold increase in cell number, showing that resveratrol helps overcome the cell cycle arrest. Of note, the polyphenol proved to be cytotoxic only at concentrations over 200 μM (Demidenko and Blagosklonny, 2009).

Resveratrol treatment in endothelial progenitor cells (EPCs) also showed prevention of replicative senescence, with reduced SA-β-Gal positive staining in comparison to controls (Xia et al., 2009). Treatment also demonstrated an increase in proliferative and migrating capabilities of EPCs, alongside a dose-dependent increase in telomerase activity, further highlighting the potential anti-senescence effects of resveratrol. This increase in telomerase activity was attributed to the activation of the PI3K-AKT pathway, which was shown to be phosphorylated in a resveratrol dose-dependent manner (Xia et al., 2009).

Moreover, when a large-scale *in vivo* study of resveratrol in genetically heterogenous (outbred) mice was conducted in parallel with rapamycin treatment, analysis of activity showed that there was no significant difference between control and resveratrol-treated mice. However, rapamycin-treated mice showed a 10% and 18% increase in median survival in males and females, respectively (Miller et al., 2010). The study highlighted the minimal effect resveratrol has on overall survival *in vivo*, even if *in vitro* studies suggested potential anti-senescence properties. In line with this, *in vivo* research from an Interventions Testing Program, which investigated the effect of administering resveratrol to a genetically heterogenous mouse model from a young age, showed no significant positive or negative effects in lifespan (Strong et al., 2012).

Nevertheless, a clinical trial involving the administration of resveratrol in older patients is underway, with participants taking a placebo, 1000 mg or 1500 mg per day in capsule form. The study will look at mitochondrial and physical function, focusing on the effects in muscle. Measures of output include levels of mitochondrial enzymes, walking speed, blood glucose, blood pressure, AMPK protein levels and resistance to muscle fatigue (REVIVE – Clinicaltrials.gov, 2019b). Despite studies and research still being conducted on resveratrol, it is also widely available to the public in tablet form, and is found in many anti-aging based cosmetics.

## Conclusion: a Long Road Ahead

With life expectancy on the increase and a greater geriatric population with chronic illnesses, management of the elderly should be focused on prevention of tissue dysfunction and maintaining a better quality of life for longer times, what is known as increasing health span. In the last decade, the field of senescence has seen the emergence of senolytic therapies, which are being investigated intensively through *in vivo* research and, recently, a move toward clinical trials. Currently, a universal standard for senescence research isn’t recognized. Different groups were able to create models *in vitro* using multiple inducers to cause DNA damage. All research should aim to test nutraceuticals first on an *in vitro* model preferably in human derived cells which have entered replicative senescence through multiple passaging. However, this kind of cell preparation would take a long time to reach senescence and only produce a finite number of cells for testing. Alternatively, using an *in vitro* mouse replicative senescence model is more feasible, as cells they stop proliferating after 5–6 passages (Khan and Gasser, 2016). Senolytics or senostatics could next be applied *in vivo* using aged wild-type mice. Despite this, multiple mouse models exist to test senolytic and senostatic application such as progeroid mice, which mimic the human syndrome known as progeria. Mice naturally reach full term within 6 months and can arise from BubR1^H/H^, Zmpste24^–/–^, Sod1^–/–^ or Ercc^–/^Δ strains (Yousefzadeh et al., 2018a).

Senolytics could be used as a preventative in the elderly, as a supplement to clear senescent cells to thus improve or maintain tissue and organ health. They are also being looked at as an adjuvant cancer therapy, with the aim of clearing treatment-induced senescent cells and thus reducing the probability of relapse (Tabasso et al., 2019). They are also being investigated in telomere biology. Telomerase is capable of reversing telomere erosion and has been targeted in peripheral blood mononuclear cells by plant derived telomerase activators (Tsoukalas et al., 2019). TA-65, a derivative of *Astragalus membranaceus*, has been shown to elongate telomerase in a telomere deficient mouse model (de Jesus et al., 2011).

Recent studies argue for a pathogenic role of senescent cells, which would contribute to a range of aging related diseases, such as osteoarthritis (Jeon et al., 2018), cardiovascular disease (Childs et al., 2018) and cataract (Fu et al., 2016). Senescent cells are found in aging related cognitive decline but also in neurodegenerative diseases such as Alzheimer’s disease and Parkinson’s disease (Baker and Petersen, 2018) which are typically identified clinically and further characterized through imaging or at autopsy. Therefore, the possible application of senolytics in a wide range of clinical scenarios is becoming an attractive concept (Paez-Ribes et al., 2019).

However, for senolytics to be widely used in aged but otherwise healthy populations to prevent tissue dysfunction, unwanted side effects have to be kept to the minimum. The use of nutraceutical based senolytics could result in fewer complications, while retaining anti-senescent effects. Despite promising *in vitro* reports, the data on the *in vivo* efficacy of nutraceutical senolytics is still sparse and, in some cases, contradictory. Thus, more research is still needed to determine whether they could be an attractive alternative to the most used chemical senolytics, such as dasatinib + quercetin, which have shown promising results in preliminary short-term clinical trials (Hickson et al., 2019).

It also has to be taken in consideration that this therapeutic field is new, and the use of nutraceuticals as senolytics may also come with its drawbacks. The potential toxicity of the compounds and their adverse interactions with existing therapies for other health issues, needs to be carefully investigated. Because of this, it is worrying that some of these compounds have reached the wider public without proper validation or complete safety studies. Caution should be exerted when dealing with them. The adoption of nutraceutical senolytics as harmless complements may need to be discouraged until more information is available.

## Author Contributions

AK researched and composed the manuscript. CS discussed the data. CS and SM edited the manuscript.

## Conflict of Interest

The authors declare that the research was conducted in the absence of any commercial or financial relationships that could be construed as a potential conflict of interest.
